# Evaluation of an Automated Screening Assay, Compared to Indirect Immunofluorescence, an Extractable Nuclear Antigen Assay, and a Line Immunoassay in a Large Cohort of Asian Patients with Antinuclear Antibody-Associated Rheumatoid Diseases: A Multicenter Retrospective Study

**DOI:** 10.1155/2018/9094217

**Published:** 2018-05-02

**Authors:** Seri Jeong, Hyunyong Hwang, Juhye Roh, Jung Eun Shim, Jinmi Kim, Geun-Tae Kim, Hee-Sang Tag, Hyon-Suk Kim

**Affiliations:** ^1^Department of Laboratory Medicine, Kosin University College of Medicine, 262 Gamcheon-ro, Seo-gu, Busan 49267, Republic of Korea; ^2^Department of Laboratory Medicine, Severance Hospital, Yonsei University College of Medicine, 50 Yonsei-ro, Seodaemun-gu, Seoul 03722, Republic of Korea; ^3^Department of Statistics, Pusan National University Hospital, 179 Gudeok-ro, Seo-gu, Busan 49241, Republic of Korea; ^4^Department of Rheumatology, Kosin University College of Medicine, 262 Gamcheon-ro, Seo-gu, Busan 49267, Republic of Korea

## Abstract

We assessed the diagnostic utility of the connective tissue disease (CTD) screen as an automated screening test, in comparison with the indirect immunofluorescence (IIF), EliA extractable nuclear antigen (ENA), and line immunoassay (LIA) for patients with antinuclear antibody- (ANA-) associated rheumatoid disease (AARD). A total of 1115 serum samples from two university hospitals were assayed using these four autoantibody-based methods. The AARD group consisted of patients with systemic lupus erythematosus (SLE), systemic sclerosis (SSc), Sjögren's syndrome (SS), and mixed connective tissue disease (MCTD). The qualitative results of all four autoantibody assays showed a significant association with AARDs, compared to controls (*P* < 0.0001 for all). The areas under the receiver operating characteristic curves (ROC-AUCs) of the CTD screen for differentiating total AARDs, SLE, SSc, SS, and MCTD from controls were 0.89, 0.93, 0.73, 0.93, and 0.95, respectively. The ROC-AUCs of combination testing with LIA were slightly higher in patients with AARDs (0.92) than those of CTD screen alone. Multivariate analysis indicated that all four autoantibody assays could independently predict AARDs. CTD screening alone and in combination with IIF, EliA ENA, and LIA are potentially valuable diagnostic approaches for predicting AARDs. Combining CTD screen with LIA might be effective for AARD patients.

## 1. Introduction

Autoantibodies are closely related to clinical manifestations or the prognosis of patients with antinuclear antibody- (ANA-) associated rheumatoid diseases (AARDs), including systemic lupus erythematosus (SLE), systemic sclerosis (SSc), Sjögren's syndrome (SS), and mixed connective tissue disease (MCTD), who generally suffer from diffuse organ damage [1, 2]. Antinuclear antibodies (ANAs), a kind of autoantibody, are directed against a variety of nuclear antigens. The detection of ANAs is useful for diagnosing patients with AARDs [3, 4]. Indirect immunofluorescence (IIF) assays with cultured human epithelial carcinoma cells (HEp-2 cells) have been regarded as a gold standard method [5]. However, IIF is a labor-intensive and time-consuming procedure and exhibits poor reproducibility due to the subjective interpretation of results [4, 6].

Enzyme immunoassays (EIAs) have been developed as alternatives to IIF for ANA screening and are widely used in clinical laboratories, enabling automation and quantitation of ANA screening [7]. The connective tissue disease (CTD) screen (Phadia AB, Uppsala, Sweden) used in this study is a recently introduced EIA-based assay employing 17 different human recombinant antigens. After the initial screen for ANAs, autoantibodies to extractable nuclear antigen (ENA) are frequently detected because of their diagnostic and prognostic significance. Identification of anti-ENA antibodies plays a critical role in the diagnosis and management of AARD [1, 8]. EliA ENA assays (Phadia AB) for detecting autoantibodies to dsDNA, U1RNP, Sm, Ro/SSA, La/SSB, Scl-70, Pm-scl, Jo-1, and CENP have been introduced in the form of several different EIA kits, and line immunoassays (LIAs) have been widely applied for confirmatory testing [9]. Little information is available regarding evaluation of the performance of these autoantibody assays simultaneously for autoantibodies and consequent antibody-disease associations. Further, most previous population studies involved patients in Europe or the USA.

In this study, we evaluated the current diagnostic performance of an automated CTD screening assay in patients with AARDs. The diagnostic utility of the assay was compared with that of the HEp-2 cell-based IIF, EliA ENA, and LIA tests in a large Asian population. We also investigated the diagnostic performance of the CTD screen in combination with the other three autoantibody assays for each AARD.

## 2. Materials and Methods

### 2.1. Study Design

A total of 1115 sera from patients who visited two university hospitals in Korea for AARD evaluation were collected to demonstrate the diagnostic performance of the CTD screen (Phadia AB, Uppsala, Sweden), as well as IIF (Fluoro HEPANA test, MBL Co., Nagoya, Japan), EliA ENA (Phadia AB), and LIA (Euroimmun AG, Lübeck, Germany) testing. The samples were collected randomly, and results from the same patients were not included repeatedly. This study was approved by the independent Institutional Review Board of Severance Hospital and Kosin University Gospel Hospital. Because residual serum samples were obtained from patients during routine screening for the detection of autoantibodies in our clinical laboratory, this study was exempted from the requirement for informed patient consent. The specimens were retrospectively classified according to predefined diagnoses as follows: total AARD (*n* = 112), SLE (*n* = 67), SSc (*n* = 21), SS (*n* = 19), MCTD (*n* = 5), and control (*n* = 1003). The total AARD value was derived from the number of patients with SLE, SSc, SS, or MCTD. The controls were consecutive patients who consulted the rheumatology clinics and for whom the rheumatologists considered it necessary to request ANA testing. After work-ups, these patients were diagnosed not to have AARDs.

All patients were diagnosed by specialized rheumatologists in the clinics of Severance Hospital and Kosin University Gospel Hospital, based on the criteria of the American College of Rheumatology and Systemic Lupus International Collaborating Clinics [10, 11] for SLE, the American College of Rheumatology/European League [12] for SSc, the American-European Consensus Classification [13] for SS, and Alarcon-Segovia and Cardiel [14] for MCTD. The control group consisted of patients with several nonsystemic rheumatic diseases, considering actual clinical laboratory status.

### 2.2. IIF for ANAs

IIF microscopy on HEp-2 cells for ANAs was performed using a commercially available Fluoro HEPANA Kit (MBL Co.), according to the manufacturer's instructions. The serum samples were diluted with phosphate-buffered saline to a 1 : 40 ratio. The procedures for IIF were conducted as described previously [15].

### 2.3. CTD Screen

Testing with the CTD screen (Phadia AB) was conducted on a Phadia 250 instrument (Phadia AB). The assays were conducted according to the manufacturer's instructions, as described before [15, 16]. The previously applied cutoffs were also used in this study; cutoffs greater than 1.0 were considered positive, ratios ranging from 0.7 to 1.0 were equivocal, and values less than 0.7 were negative.

### 2.4. EliA ENA

Nine kinds of EliA ENA assays for detecting autoantibodies against dsDNA, U1RNP, Sm, Ro/SSA, La/SSB, Scl-70, Pm-scl, Jo-1, and CENP were performed using the respective EliA ENA kits (Phadia AB) on a Phadia 250 instrument (Phadia AB). All procedures were conducted as indicated in the manufacturer's instructions. The procedures followed were similar to those described above for the CTD screen. The cutoff value for dsDNA was 15 IU/ml, and that for U1RNP, Sm, Ro/SSA, La/SSB, Scl-70, Pm-scl, Jo-1, and CENP was 10 U/ml.

### 2.5. LIA

The EUROLINE test (Euroimmun AG) enabled detection of 14 autoantibodies against RNP, Sm, Ro/SSA60, Ro/SSA52, La/SSB, Scl-70, Pm-Scl, Jo-1, CENP, PCNA, nucleosomes, histones, ribosomal-P, and AMA-M2. These antigens were detected as discrete lines on a nylon membrane with a plastic backing. These strips were incubated sequentially with sera, alkaline phosphatase-labeled anti-human IgG antibodies, and substrate solution (containing nitroblue tetrazolium chloride and 5-bromo-4-chloro-3-indolyl phosphate), according to the manufacturer's instructions. Reaction intensities were automatically interpreted using Eurolinescan software (Euroimmun AG), similar to the procedures described in a previous report [17].

### 2.6. Statistical Analysis

Statistical analyses were performed using PASW software, version 24.0 (formerly, SPSS Statistics) (SPSS Inc., Chicago, IL, USA) and Analyse-it Method Evaluation Edition software, version 2.26 (Analyse-it Software Ltd., Leeds, UK). Comparisons using chi-squared test and the Mann–Whitney *U* test were performed as described before [15]. Cohen's kappa coefficients were calculated to estimate the agreement among the results of the CTD screen and the other three autoantibody assays, and McNemar's test was also conducted. Receiver operating characteristic (ROC) curves were plotted for data from the CTD screen alone or in combination with the other assays, in order to assess their diagnostic abilities to distinguish between the AARD groups and the control group. The areas under the ROC curves (ROC-AUCs) for the CTD screen and its combination with the other three assays were compared. Binary logistic regression analysis was performed to estimate the ROC-AUCs for IIF and CTD screen combinations, similar to the previously described method [15, 18]. Multivariate logistic regression analysis was performed by setting the presence of an AARD as the dependent variable and age, sex, and the results of IIF, CTD screen, EliA ENA, and LIA testing as covariables. *P* values less than 0.05 were considered statistically significant.

## 3. Results

### 3.1. Study Population Characteristics

The basic characteristics of the study population are presented in Table 1. The median ages of patients in the total AARD and control groups were 39.0 and 50.0 years, respectively (*P* < 0.0001). The proportion of female patients between the total AARD and control group was not significant (97.3% versus 99.2%, *P* = 0.0561).

### 3.2. Comparison of the CTD Screen, IIF, EliA ENA, and LIA Results according to Predefined AARD Criteria

The qualitative results of the CTD screen, IIF, EliA ENA, and LIA tests in patients with predefined AARDs and control subjects are summarized in Table 1. The results from all four assays were significantly associated with the total AARD group, compared to the control group (*P* < 0.0001 for all) (Table 1).

### 3.3. Overall Agreement among the CTD Screen, IIF, EliA ENA, and LIA Test Results according to the AARD Groups

An overall comparison of the results, including the concordance rates, kappa coefficient, and *P* values of McNemar testing, among the four autoantibody assays is presented in Table 2.

#### 3.3.1. Concordance Rates

When the CTD screen ratios were considered as qualitative results with a cutoff of 1.0 (equivocal ratios scored as negative results), as suggested by the manufacturer, the overall concordance rates were 80.4% for the CTD screen versus IIF, 89.3% versus EliA ENA, and 79.5% versus LIA in the total AARD group. In addition, the overall concordance rates were 78.6% for the CTD screen with a cutoff of 0.7 (equivocal ratios scored as positive results) versus IIF, 89.3% versus EliA ENA, and 81.3% versus LIA.

#### 3.3.2. Kappa Coefficients

The kappa coefficients between the CTD screen and EliA ENA were good (0.70), while those between the CTD screen and IIF were moderate (0.47 for cutoff 1.0 and 0.41 for cutoff 0.7), and those between the CTD screen and LIA were fair (0.29 for cutoff 1.0 and 0.32 for cutoff 0.7).

#### 3.3.3. McNemar Test

The *P* values of McNemar tests showed no statistically significant differences between the CTD screen and LIA (0.6776 for cutoff 1.0 and 1.0000 for cutoff 0.7), whereas the differences between the CTD screen versus IIF (0.0169 for cutoff 1.0 and 0.0066 for cut-off 0.7) and CTD screen versus EliA ENA (0.0063 for cutoff 1.0 and 0.0005 for cutoff 0.7) were significant in the total AARD group.

### 3.4. Diagnostic Performances of the CTD Screen, IIF, EliA ENA, and LIA Tests

The calculated ROC-AUCs, sensitivities, specificities at the best cutoffs, likelihood ratios, and odds ratios are shown in Table 3.

#### 3.4.1. ROC-AUCs

**Figure 1:**
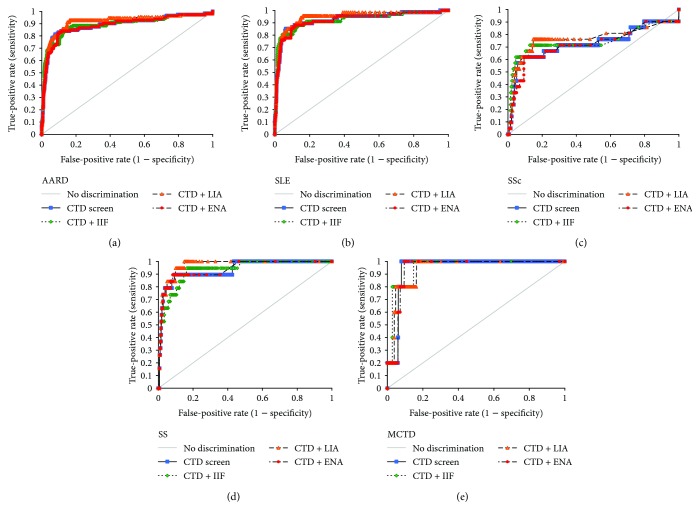
Diagnostic performance of the CTD screen, tested independently and in combination with autoantibody assays. (a) Receiver operating characteristic (ROC) curves of the CTD screen (0.89) and its combination with autoantibody assays for discriminating total antinuclear antibody-associated rheumatoid disease patients (*n* = 112) from control subjects (*n* = 1003); (b) ROC curves for the CTD screen (0.93) and its combination with autoantibody assays, differentiating patients with systemic lupus erythematosus (SLE) (*n* = 67) from control subjects; (c) ROC curves for the CTD screen (0.73) combined with autoantibody assays, differentiating patients with systemic sclerosis (SSc) (*n* = 21) from control subjects; (d) ROC curves for the CTD screen (0.93) and its combination with autoantibody assays, discriminating patients with Sjögren's syndrome (SS) (*n* = 19) from control subjects; (e) ROC curves for the CTD screen (0.95) and its combination with autoantibody assays, differentiating patients with mixed connective tissue disease (MCTD) (*n* = 5) from control subjects.

The best cutoff was determined when the sum of sensitivity and specificity was maximized. When these cut-off values were applied, the sensitivities of the CTD screen and its combination with IIF, EliA ENA, and LIA were 81.3%, 85.7%, 83.0%, and 92.9%, respectively, and the specificities were 92.1%, 85.2%, 89.6%, and 83.7%, respectively, in the total AARD group. The ROC-AUCs of the CTD screen and its combination with IIF, EliA ENA, and LIA were over 0.90 for all subgroups, indicating excellent diagnostic performances, except for the ROC-AUCs in the SSc group, which showed fair performance (Figure 1). The differences between the ROC curves of the CTD screen and its combinations when differentiating AARDs from the control group were also analyzed. The ROC-AUCs for CTD screen and its combination with LIA were 0.89 and 0.92, respectively, demonstrating a significant difference (*P* = 0.0267) in the total AARD group, while the other combinations in the total AARD group, as well as in the SLE, SSc, SS, and MCTD groups, did not show significant differences.

#### 3.4.2. Likelihood Ratio and Odds Ratio

The positive and negative likelihood ratios of the CTD screen and its combination with IIF, EliA ENA, and LIA in the total AARD, SLE, SS, and MCTD groups were considered very useful (positive likelihood ratio > 5.0 or ≤0.2 for all groups). The positive likelihood ratios for the CTD screen and its combination with IIF, EliA ENA, and LIA in the SSc group were also very useful, while the negative likelihood ratios among them ranged from 0.3 to 0.4, indicating that they were just useful, based on the criteria of Solomon et al. [4]. Additionally, the odds ratios of CTD screen with LIA (67.0) were marginally higher than those of CTD screen alone (50.7) in the total AARD group.

### 3.5. Multivariate Analysis of CTD Screen, IIF, EliA ENA, and LIA Test Results

Multivariate analysis was performed using the presence of an AARD as the binary dependent variable and age and sex of the patient and the CTD screen, IIF, EliA ENA, and LIA results as predictors. We found that age and the results from CTD screen, IIF, EliA ENA, and LIA testing were independently associated with total AARD (Table 4). Our results showed that age, CTD screen, IIF, and LIA results for SLE; age, sex, IIF, and LIA results for SSc; and EliA ENA and LIA results for SS independently correlated with the respective rheumatic diseases. Sex was not a predictor in the SS and MCTD groups in the multivariate analysis because all subjects were female.

## 4. Discussion

Diagnostic applications of the CTD screen was assessed in Korean patients with AARDs, retrospectively. The performance of an automated screening assay was compared to HEp-2 cell-based IIF, EliA ENA, and LIA methods. In addition, diagnostic values of combining the CTD screen and these 3 autoantibody assays were investigated for each AARD.

The proportions of CTD screen, IIF, EliA ENA, and LIA positivities were higher in patients diagnosed with AARDs than in the control group, indicating that these four assays would be useful for detecting the patients with AARDs. Previous reports have shown similar results with these assays [19–22]. The sensitivities and specificities of these four assays would be affected because the classification criteria of each AARD were applied to our study as a standard. Regarding the sensitivity of the HEp-2 IIF, the values in this study were considerably low when compared to those in the previous reports [23, 24]. Meanwhile, there are several studies with decreased sensitivities of HEp-2 IIF such as 69.0%, 36.4%, and 53.7% [16, 22, 25], even lower than those of our study. Although our laboratory obtained accreditation, tested kits, condition of microscopes, interobserver variations, and low number of the patient population may have an influence on the values, similar to these studies with lowered sensitivities.

The negative results in the AARD groups were caused by earlier clinical observations and subsequent development of positivity in follow-up or a negative conversion after initial positivity at presentation because of immunosuppressive treatment, based on a review of each medical chart. Clinical and serological presentations can fluctuate over time in AARD patients, according to the results of several studies [19, 26].

Regarding the overall concordance rates between the CTD screen and IIF test, the agreements ranged from 87% to 95% when comparing the EIA assays and IIF in a previous study [27]. Previously, Cohen's kappa value observed when combining EIA results of ENA and IIF tests was reported to be 0.30 [19], which was the same as that found with combined EliA ENA and IIF testing (0.30) and lower than that of the CTD screen and the IIF test (0.70), in this study. The previous report showed that the EIA for ENA and IIF results exhibited fair agreement for patients with SLE and that the kappa agreement for patients with scleroderma was good. Our results also demonstrated that the kappa value between EliA ENA and IIF of the SSc group (0.45) was higher than that of the SLE group (0.34). The kappa values between the CTD screen and IIF test were higher than those of EliA ENA and IIF in all subgroups, indicating that the CTD screen was more appropriate for screening with more diverse antigens than the EliA ENA test.

With respect to the LIA results, the kappa coefficients compared to IIF were similar to the previous results in the total AARD group (0.45 versus 0.404), as well as the SLE (0.28 versus 0.298) and SSc (0.48 versus 0.573) groups [28]. These agreements between the LIA and IIF methods were also similar to those between the LIA test and CTD screen, reflecting the characteristics of CTD screen and IIF as screening assays. There were slight differences between the different study populations and the cutoff used for each method. A detailed description of the agreements found between these tests (including concordance rates, Cohen's kappa values, and *P* values for McNemar tests) among the widely used autoantibody assays has been scarcely reported in the literature with large groups.

The diagnostic abilities of the CTD screen to discriminate each AARD subgroup from the control group were analyzed because the clinical utility of the CTD screen can vary with the characteristics of AARD patients and nonrheumatic conditions. The portion of SSc patients in the total AARD group may explain the lower CTD screen performance in the total AARD group, based on the ROC-AUCs. A previous study was conducted to evaluate automated ENA screening assay with 9 antigens and showed an ROC-AUC of 0.823, sensitivity of 63%, and specificity of 91% for AARD patients [22], which were slightly lower than the values obtained in our study. Another previous report showed that the sensitivities of the CTD screen for SLE, SSc, SS, and MCTD groups were 74%, 72%, 89%, and 100%, respectively [20]. Although the sensitivities reported for previous studies and those of our study were slightly different, the highest sensitivity for the MCTD group and lowest sensitivity for the SSc group [20] were concordant to our study. With respect to the cutoffs, we described the CTD screen results at 0.7 and 1.0 because these values are suggested by the manufacturer's instructions. Based on the best cutoff of ROC-AUCs in our study, 1.0 of CTD screen is closer to it than 0.7 for total AARD, SLE, SSc, and MCTD and vice versa for SS. Therefore, 1.0 would be the best cutoff for patients with AARD, including SLE, SSc, and MCTD while 0.7 would be more appropriate for patients with SS. After verification, applying the optimal cutoff for each clinical laboratory is recommended.

Regarding clinical implications based on our data, CTD screen was comparable to IIF or the other assays because the 95% confidence intervals (CIs) of sensitivities and ROC-AUCs were overlapped with those in the others, indicating no statistical significance. Further, CTD screen exhibited slightly better specificity than the other assays according to the results of Tables 1 and 3, raising the possibility of an alternative screening assay. However, sufficient review of previous literatures for ANA testing [5, 29] is necessary when these assays are adopted to individual clinical laboratories. A variety of confounding factors such as patient populations, controls, applied methods, and cutoffs might affect the analyzed results.

The positive likelihood ratios of the CTD screen were very useful and conclusive, with respect to pretest and posttest probabilities [4, 30]. Previously published data also demonstrated positive results for the CTD screen at a cutoff of 1.0, with positive likelihood ratios greater than 5.0 in the SLE, SSc, SS, and MCTD groups [20]. The negative likelihood ratios for the CTD screen were less than 0.2 with the exception of SSc patients. Op De Beeck et al. [20] showed a similar pattern for the CTD screen in patients with SLE (0.3), SSc (0.3), and SS (0.1), which was considered to be very useful or useful tests, based on the negative likelihood ratios [4].

Based on the values of ROC-AUCs and odds ratios, concurrent determination with the CTD screen and LIA test demonstrated slightly improved diagnostic utilities in patients in the total AARD group, compared to the CTD screen alone. According to a previous report, the diagnostic performance of the CTD screen was significantly improved when used in combination with IIF, based on the likelihood ratios and ROC-AUCs [31]. The study population was patients of the university hospital in Belgium, and the results were slightly varied according to the diagnosis, suggesting different strategies for each AARDs. Therefore, uniformed and simultaneous screening by the CTD screen and IIF [29] would not be effective in cases with strong clinical manifestations associated with each AARD. Further, investigating laboratory findings with respect to other immunologic disorders might be essential for diagnosing each AARD when the screening assay shows positive results.

Multivariate analysis was also conducted for age and sex to assess CTD screen, IIF, EliA ENA, and LIA results as predictor variables for AARD groups and to control for potential confounding factors. ANAs were previously found in healthy individuals, particularly women older than 40 years [32]. Therefore, the factors of age and sex were incorporated into our multivariate model. The multivariate analysis results revealed that CTD screen, IIF, EliA ENA, and LIA results were independently related to the total AARD group, indicating that these four autoantibody assays could be applied for diagnosing AARDs. However, not all assays showed significant *P* values in the subgroups. Based on our multivariate analysis, a combination with LIA for diagnostic work-ups of patients with SSc or SS would be necessary, when the CTD screen was applied to the clinical laboratories.

The profiles for the individual autoantibodies in the EliA ENA and LIA tests were generally concordant with previous reports [17, 28]. The high specificities of EliA ENA and LIA for individual autoantibodies (over 95.0%) were similar to those of a previous study, which showed at least 97% specificities [17]. These assays, especially the LIA, have been used as confirmatory tests, based on their high specificities [9]. The relatively low specificities of anti-Ro/SSA and Scl-70, which are related to SS and SSc, respectively [17, 24], might contribute to the lowered specificities of LIA.

Our study design has some limitations in that most of our study population was composed of females, especially the SS and MCTD groups. Among the hospitalized SLE patients, 165 out of 180 (91.8%) were female, based on the information in the Korean lupus network registry [33]. Although our data reflected a predominant portion of female patients, further studies involving male patients should be followed. Moreover, the number of patient population was small, especially MCTD, because of low prevalence. Further studies involving large study populations are necessary, considering the statistical power, interpretation, and application of the results to AARD patients.

## 5. Conclusions

In conclusion, we evaluated the diagnostic utilities of CTD screen, IIF, EliA ENA, and LIA testing to predict AARDs. Although a few published reports have discussed the usefulness of the CTD screen alone, no report has assessed a large population of Korean patients in Asia or the diagnostic performance of these frequently used autoantibody assays for AARDs in current use. CTD screening and the other three assays are potentially valuable diagnostic tools for detecting AARDs. In particular, the CTD screen showed considerable diagnostic performance for patients with SLE rather than SSc, and its combination with the LIA test might be more effective for diagnosing total AARDs than the CTD screen alone. Our results provided recent information regarding the CTD screen, IIF, EliA ENA, and LIA methods for testing patients with AARDs to facilitate appropriate patient management, with cautious clinical impressions of patients' diagnosis.

## Figures and Tables

**Table 1:** Demographic characteristics of the study population and the qualitative values for four autoantibody assays.

Variables		Total AARD (*n* = 112)	SLE (*n* = 67)	SSc (*n* = 21)	SS (*n* = 19)	MCTD (*n* = 5)	Control (*n* = 1003)	*P* value^∗^
Age (years)^∗∗^		39.0 (23.0–51.0)	37.0 (17.2–44.8)	39.0 (26.7–49.7)	54.0 (42.0–62.0)	43.0 (35.0–47.3)	50.0 (37.0–61.0)	<0.0001
Number of females, %		109, 97.3	66, 98.5	19, 90.5	19, 100.0	5, 100.0	995, 99.2	0.0561
CTD screen, cutoff ratio 0.7 (number), %	Positive	93, 83.0	58, 86.6	13, 61.9	17, 89.5	5, 100.0	100, 10.0	<0.0001
	Negative	19, 17.0	9, 13.4	8, 38.1	2, 10.5	0, 0.0	903, 90.0	
CTD screen, cutoff ratio 1.0 (number), %	Positive	91, 81.3	57, 85.1	13, 61.9	16, 84.2	5, 100.0	79, 7.9	<0.0001
	Negative	21, 18.7	10, 14.9	8, 38.1	3, 15.8	0, 0.0.	924, 92.1	
HEp-2 IIF (number), %	Positive	79, 70.5	51, 76.1	13, 61.9	11, 57.9	4, 80.0	99, 9.9	<0.0001
	Negative	33, 29.5	16, 23.9	8, 38.1	8, 42.1	1, 20.0	904, 90.1	
EliA ENA (number), %	Positive	81, 72.3	51, 76.1	9, 42.9	17, 89.5	4, 80.0	93, 9.3	<0.0001
	Negative	31, 27.7	16, 23.9	12, 57.1	2, 10.5	1, 20.0	910, 90.7	
LIA (number), %	Positive	94, 83.9	58, 86.6	14, 66.7	18, 94.7	4, 80.0	141, 14.1	<0.0001
	Negative	18, 16.1	9, 13.4	7, 33.3	1, 5.3	1, 20.0	862, 85.9	

AARD: antinuclear antibody-associated rheumatoid disease; SLE: systemic lupus erythematosus; SSc: systemic sclerosis; SS: Sjögren's syndrome; MCTD: mixed connective tissue disease; IIF: indirect immunofluorescence; ENA: extractable nuclear antigen; LIA: line immunoassay. ^∗^Chi-squared test for nominal variables and Mann–Whitney *U* test for continuous variables in the total AARD group versus control. ^∗∗^Data are expressed as the median (1st to 3rd quartiles).

**Table 2:** Overall agreements among the four autoantibody assays, according to the AARD groups.

Group (*n*)	Assays	CTD screen, cutoff ratio 0.7			CTD screen, cutoff ratio 1.0			HEp-2 IIF			EliA ENA			LIA		
		Concordance (%)	Kappa^∗^	McNemar^∗∗^	Concordance (%)	Kappa^∗^	McNemar^∗∗^	Concordance (%)	Kappa^∗^	McNemar^∗∗^	Concordance (%)	Kappa^∗^	McNemar^∗∗^	Concordance (%)	Kappa^∗^	McNemar^∗∗^
Total AARD (*n* = 112)	CTD screen, cutoff ratio 0.7	—	—	—	98.2	0.94 (0.86–1.02)	0.5000	78.6	0.41 (0.22–0.60)	0.0066	89.3	0.70 (0.54–0.85)	0.0005	81.3	0.32 (0.09–0.55)	1.0000
	CTD screen, cutoff ratio 1.0	98.2	0.94 (0.86–1.02)	0.5000	—	—	—	80.4	0.47 (0.29–0.65)	0.0169	89.3	0.70 (0.55–0.86)	0.0063	79.5	0.29 (0.07–0.51)	0.6776
	HEp-2 IIF	78.6	0.41 (0.22–0.60)	0.0066	80.4	0.47 (0.29–0.65)	0.0169	—	—	—	71.4	0.30 (0.11–0.49)	0.8601	74.1	0.28 (0.09–0.47)	0.0081
	EliA ENA	89.3	0.70 (0.54–0.85)	0.0005	89.3	0.70 (0.55–0.86)	0.0063	71.4	0.30 (0.11–0.49)	0.8601	—	—	—	75.9	0.31 (0.11–0.50)	0.0192
	LIA	81.3	0.32 (0.09–0.55)	1.0000	79.5	0.29 (0.07–0.51)	0.6776	74.1	0.28 (0.09–0.47)	0.0081	75.9	0.31 (0.11–0.50)	0.0192	—	—	—
SLE (*n* = 67)	CTD screen, cutoff ratio 0.7	—	—	—	98.5	0.94 (0.82–1.06)	1.0000	83.6	0.47 (0.21–0.73)	0.0654	89.6	0.66 (0.44–0.88)	0.0156	82.1	0.23 (−0.08 to 0.54)	1.0000
	CTD screen, cutoff ratio 1.0	98.5	0.94 (0.82–1.06)	1.0000	—	—	—	85.1	0.53 (0.28–0.78)	0.1094	91.0	0.72 (0.51–0.92)	0.0313	80.6	0.20 (−0.10 to 0.50)	1.0000
	HEp-2 IIF	83.6	0.47 (0.21–0.73)	0.0654	85.1	0.53 (0.28–0.78)	0.1094	—	—	—	76.1	0.34 (0.08–0.60)	1.0000	77.6	0.28 (0.01–0.54)	0.1185
	EliA ENA	89.6	0.66 (0.44–0.88)	0.0156	91.0	0.72 (0.51–0.92)	0.0313	76.1	0.34 (0.08–0.60)	1.0000	—	—	—	77.6	0.28 (0.01–0.54)	0.1185
	LIA	82.1	0.23 (−0.08 to 0.54)	1.0000	80.6	0.20 (−0.10 to 0.50)	1.0000	77.6	0.28 (0.01–0.54)	0.1185	77.6	0.28 (0.01–0.54)	0.1185	—	—	—
SSc (*n* = 21)	CTD screen, cutoff ratio 0.7	—	—	—	100.0	1.00 (1.00-1.00)	1.0000	81.0	0.60 (0.24–0.95)	1.0000	81.0	0.63 (0.33–0.93)	0.1250	76.2	0.48 (0.09–0.87)	1.0000
	CTD screen, cutoff ratio 1.0	100.0	1.00 (1.00-1.00)	1.0000	—	—	—	81.0	0.60 (0.24–0.95)	1.0000	81.0	0.63 (0.33–0.93)	0.1250	76.2	0.48 (0.09–0.87)	1.0000
	HEp-2 IIF	81.0	0.60 (0.24–0.95)	1.0000	81.0	0.60 (0.24–0.95)	1.0000	—	—	—	71.4	0.45 (0.10–0.80)	0.2188	76.2	0.48 (0.09–0.87)	1.0000
	EliA ENA	81.0	0.63 (0.33–0.93)	0.1250	81.0	0.63 (0.33–0.93)	0.1250	71.4	0.45 (0.10–0.80)	0.2188	—	—	—	66.7	0.36 (0.02–0.71)	0.1250
	LIA	76.2	0.48 (0.09–0.87)	1.0000	76.2	0.48 (0.09–0.87)	1.0000	76.2	0.48 (0.09–0.87)	1.0000	66.7	0.36 (0.02–0.71)	0.1250	—	—	—
SS (*n* = 19)	CTD screen, cutoff ratio 0.7	—	—	—	94.7	0.77 (0.35–1.20)	1.0000	57.9	0.04 (−0.28 to 0.36)	0.0703	100.0	1.00 (1.00-1.00)	1.0000	84.2	−0.08 (−0.18 to 0.03)	1.0000
	CTD screen, cutoff ratio 1.0	94.7	0.77 (0.35–1.20)	1.0000	—	—	—	63.2	0.17 (−0.20 to 0.55)	0.1250	94.7	0.77 (0.35–1.20)	1.0000	78.9	−0.09 (−0.22 to 0.05)	0.6250
	HEp-2 IIF	57.9	0.04 (−0.28 to 0.36)	0.0703	63.2	0.17 (−0.20 to 0.55)	0.1250	—	—	—	57.9	0.04 (−0.28 to 0.36)	0.0703	63.2	0.14 (−0.12 to 0.40)	0.0156
	EliA ENA	100.0	1.00 (1.00-1.00)	1.0000	94.7	0.77 (0.35–1.20)	1.0000	57.9	0.04 (−0.28 to 0.36)	0.0703	—	—	—	84.2	−0.08 (−0.18 to 0.03)	1.0000
	LIA	84.2	−0.08 (−0.18 to 0.03)	1.0000	78.9	−0.09 (−0.22 to 0.05)	0.6250	63.2	0.14 (−0.12 to 0.40)	0.0156	84.2	−0.08 (−0.18 to 0.03)	1.0000	—	—	—
MCT (*n* = 5)	CTD screen, cutoff ratio 0.7	—	—	—	—	—	—	—	—	—	—	—	—	—	—	—
	CTD screen, cutoff ratio 1.0	—	—	—	—	—	—	—	—	—	—	—	—	—	—	—
	HEp-2 IIF	—	—	—	—	—	—	—	—	—	—	—	—	—	—	—
	EliA ENA	—	—	—	—	—	—	60	−0.25 (−0.59 to 0.09)	1.0000	—	—	—	—	—	—
	LIA	—	—	—	—	—	—	60	−0.25 (−0.59 to 0.09)	1.0000	60	−0.25 (−0.59 to 0.09)	1.0000	—	—	—
Total (*n* = 1115)	CTD screen, cutoff ratio 0.7	—	—	—	97.9	0.92 (0.89–0.95)	<0.0001	85.6	0.48 (0.41–0.55)	0.2698	97.6	0.91 (0.88–0.94)	0.0003	85.7	0.54 (0.48–0.60)	0.0011
	CTD screen, cutoff ratio 1.0	97.9	0.92 (0.89–0.95)	<0.0001	—	—	—	87.4	0.52 (0.45–0.59)	0.5543	96.4	0.86 (0.82–0.90)	—	86.5	0.55 (0.48–0.61)	<0.0001
	HEp-2 IIF	85.6	0.48 (0.41–0.55)	0.2698	87.4	0.52 (0.45–0.59)	0.5543	—	—	—	85.3	0.45 (0.38–0.52)	0.8149	83.4	0.45 (0.39–0.52)	<0.0001
	EliA ENA	97.6	0.91 (0.88–0.94)	0.0003	96.4	0.86 (0.82–0.90)	0.6358	85.3	0.45 (0.38–0.52)	0.8149	—	—	—	85.7	0.53 (0.46–0.59)	<0.0001
	LIA	85.7	0.54 (0.48–0.60)	0.0011	86.5	0.55 (0.48–0.61)	<0.0001	83.4	0.45 (0.39–0.52)	<0.0001	85.7	0.53 (0.46–0.59)	<0.0001	—	—	—

IIF: indirect immunofluorescence; ENA: extractable nuclear antigen; LIA: line immunoassay; AARD: antinuclear antibody-associated rheumatoid disease; SLE: systemic lupus erythematosus; SSc: systemic sclerosis; SS: Sjögren's syndrome; MCTD: mixed connective tissue disease; NS: not stated because of impractical calculation. ^∗^Kappa coefficient between the assays are shown (95% confidence interval). ^∗∗^*P* values of the McNemar tests.

**Table 3:** ROC-AUC, sensitivity, and specificity of the CTD screen, tested independently and in combination.

Predefined diseases	Parameter	CTD screen	CTD screen + IIF	CTD screen + EliA ENA	CTD screen + LIA
Total AARD (*n* = 112)	ROC-AUC^∗^	0.89 (0.85–0.93)	0.89 (0.85–0.93)	0.89 (0.85–0.93)	0.92 (0.88–0.95)
	*P* value^∗^	<0.0001	<0.0001	<0.0001	<0.0001
	Sensitivity (%)^∗^	81.3 (72.8–88.0)	85.7 (77.8–91.6)	83.0 (74.8–89.5)	92.9 (86.4–96.9)
	Specificity (%)^∗^	92.1 (90.3–93.7)	85.2 (82.9–87.4)	89.6 (87.6–91.4)	83.7 (81.3–86.0)
	+LR	10.3	5.8	8.0	5.7
	−LR	0.2	0.2	0.2	0.1
	Odds ratio	50.7 (29.9–85.9)	34.7 (19.9–60.5)	42.3 (24.8–72.1)	67.0 (32.0–140.2)
SLE (*n* = 67)	ROC-AUC^∗^	0.93 (0.88–0.97)	0.92 (0.88–0.97)	0.92 (0.88–0.96)	0.95 (0.91–0.98)
	*P* value^∗^	<0.0001	<0.0001	<0.0001	<0.0001
	Sensitivity (%)^∗^	85.1 (74.3–92.6)	80.6 (69.1–89.2)	88.1 (77.8–94.7)	95.5 (87.5–99.1)
	Specificity (%)^∗^	93.7 (92.0–95.1)	95.7 (94.3–96.9)	88.3 (86.2–90.3)	83.8 (81.4–86.1)
	+LR	13.5	18.8	7.6	5.9
	−LR	0.2	0.2	0.1	0.1
	Odds ratio	85.0 (41.4–174.5)	92.7 (47.1–182.7)	55.8 (26.0–119.8)	110.7 (34.4–356.8)
SSc (*n* = 21)	ROC-AUC^∗^	0.73 (0.58–0.88)	0.75 (0.59–0.90)	0.72 (0.57–0.87)	0.76 (0.61–0.91)
	*P* value^∗^	0.0017	0.0011	0.0018	0.0003
	Sensitivity (%)^∗^	61.9 (38.4–81.9)	71.4 (47.8–88.7)	61.9 (38.4–81.9)	76.2 (52.8–91.8)
	Specificity (%)^∗^	92.5 (90.7–94.1)	87.2 (85.0–89.2)	90.6 (88.7–92.4)	85.2 (82.9–87.4)
	+LR	8.3	5.6	6.6	5.2
	−LR	0.4	0.3	0.4	0.3
	Odds ratio	20.1 (8.1–50.0)	17.1 (6.5–44.8)	15.7 (6.4–38.9)	18.5 (6.7–51.2)
SS (*n* = 19)	ROC-AUC^∗^	0.93 (0.87–0.99)	0.93 (0.88–0.98)	0.94 (0.88–0.99)	0.97 (0.95–0.99)
	*P* value^∗^	<0.0001	<0.0001	<0.0001	<0.0001
	Sensitivity (%)^∗^	89.5 (66.9–98.7)	94.7 (74.0–99.9)	89.5 (66.9–98.7)	100.0 (82.4–100.0)
	Specificity (%)^∗^	90.7 (88.8–92.5)	83.7 (81.3–86.0)	91.7 (89.8–93.4)	85.1 (82.8–87.3)
	+LR	9.7	5.8	10.8	6.7
	−LR	0.1	0.1	0.1	0.0
	Odds ratio	83.2 (18.9–365.6)	92.8 (12.3–699.7)	94.2 (21.4–414.8)	NC
MCTD (*n* = 5)	ROC-AUC^∗^	0.95 (0.92–0.98)	0.95 (0.90–100.0)	0.94 (0.91–0.98)	0.94 (0.89–100.0)
	*P* value^∗^	<0.0001	<0.0001	<0.0001	<0.0001
	Sensitivity (%)^∗^	100.0 (47.8–100.0)	100.0 (47.8–100.0)	100.0 (47.8–100.0)	100.0 (47.8–100.0)
	Specificity (%)^∗^	92.1 (90.3–93.7)	85.2 (82.9–87.4)	90.5 (88.5–92.3)	83.5 (81.1–85.8)
	+LR	12.7	6.8	10.6	6.1
	−LR	0.0	0.0	0.0	0.0
	Odds ratio	NC	NC	NC	NC

ROC-AUC: areas under the receiver operating characteristic curve; IIF: indirect immunofluorescence; ENA: extractable nuclear antigen; LIA: line immunoassay; LR: likelihood ratio; AARD: antinuclear antibody-associated rheumatoid disease; SLE: systemic lupus erythematosus; SSc: systemic sclerosis; SS: Sjögren's syndrome; MCTD: mixed connective tissue disease; NC: not calculated. ^∗^Data are shown with the 95% confidence interval in parentheses, and the *P* values presented for the ROC-AUCs were greater than 0.5.

**Table 4:** Multivariate analysis^∗^ of the outcomes of antinuclear antibody-associated rheumatoid disease.

Dependent variables	Covariate	OR	SE	95% CI	*P* value
Total AARD (*n* = 112)	Age (years)	0.9784	0.0081	0.9631 to 0.9940	0.0069
	Female sex	0.3754	0.8086	0.0769 to 1.8311	0.2256
	CTD screen	1.1017	0.0306	1.0374 to 1.1699	0.0016
	IIF	4.2961	0.2977	2.3972 to 7.6993	<0.0001
	EliA ENA	2.8036	0.3589	1.3875 to 5.6653	0.0041
	LIA	7.6024	0.3342	3.9493 to 14.6345	<0.0001
SLE (*n* = 67)	Age (years)	0.9680	0.0103	0.9485 to 0.9878	0.0017
	Female sex	0.7809	1.1675	0.0792 to 7.6986	0.8323
	CTD screen	1.1309	0.0336	1.0588 to 1.2080	0.0003
	IIF	4.5804	0.3904	2.1309 to 9.8456	0.0001
	EliA ENA	2.0365	0.4773	0.7991 to 5.1899	0.1362
	LIA	8.7782	0.4639	3.5360 to 21.7918	<0.0001
SSc (*n* = 21)	Age (years)	0.9688	0.0143	0.9420 to 0.9963	0.0265
	Female sex	0.0707	0.9631	0.0107 to 0.4669	0.0265
	CTD screen	0.9501	0.0668	0.8336 to 1.0830	0.4435
	IIF	7.6505	0.5496	2.6051 to 22.4672	0.0002
	EliA ENA	1.8686	0.6595	0.5130 to 6.8058	0.3431
	LIA	4.7949	0.5912	1.5049 to 15.2774	0.0080
SS^∗∗^ (*n* = 19)	Age (years)	1.0273	0.0163	0.9950 to 1.0606	0.0985
	CTD screen	1.0595	0.0489	0.9626 to 1.1661	0.2374
	IIF	1.8878	0.6031	0.5789 to 6.1559	0.2920
	EliA ENA	12.7179	0.8660	2.3295 to 69.4327	0.0033
	LIA	22.8184	1.1071	2.6055 to 199.8360	0.0047
MCTD^∗∗^ (*n* = 5)	Age (years)	0.9947	0.0277	0.9421 to 1.0502	0.8466
	CTD screen	1.0226	0.0629	0.9039 to 1.1569	0.7221
	IIF	8.4246	1.2892	0.6733 to 105.4167	0.0983
	EliA ENA	7.6465	1.3879	0.5036 to 116.1116	0.1427
	LIA	3.5924	1.3567	0.2515 to 51.3162	0.3459

OR: odds ratio; SE: standard error; CI: confidence interval; IIF: indirect immunofluorescence; ENA: extractable nuclear antigen; LIA: line immunoassay; AARD: antinuclear antibody-associated rheumatoid disease; SLE: systemic lupus erythematosus; SSc: systemic sclerosis; SS: Sjögren's syndrome; MCTD: mixed connective tissue disease. ^∗^Multivariate analysis was performed using the presence of each AARD as a binary dependent variable and with the age, patient sex, and the results of CTD screen, IIF, EliA ENA, and LIA tests as covariates. ^∗∗^The SS group and MCTD group were composed of females only.
